# A Subset of Nuclear Receptors are Uniquely Expressed in Uveal Melanoma Cells

**DOI:** 10.3389/fendo.2015.00093

**Published:** 2015-07-07

**Authors:** Kenneth Edward Huffman, Ryan Carstens, Elisabeth D. Martinez

**Affiliations:** ^1^Hamon Center for Therapeutic Oncology Research, Dallas, TX, USA; ^2^Department of Pharmacology, University of Texas Southwestern Medical Center, Dallas, TX, USA

**Keywords:** nuclear receptor expression, uveal melanoma, cutaneous melanoma, NCI-60, profiling

## Abstract

Uveal melanoma (UM) is recognized as the most common intraocular malignancy and the second most common form of melanoma. Nearly 50% of UM patients develop untreatable and fatal metastases. The 48-member nuclear receptor (NR) superfamily represents a therapeutically targetable group of transcription factors known for their regulation of key cancer pathways in numerous tumor types. Here, we profiled the expression of the 48 human NRs by qRT-PCR across a melanoma cell line panel including 5 UM lines, 9 cutaneous melanoma (CM) lines, and normal primary melanocytes. NR expression patterns identified a few key features. First, in agreement with our past studies identifying RXRg as a CM-specific marker, we found that UM cells also exhibit high levels of RXRg expression, making it a universal biomarker for melanoma tumors. Second, we found that LXRb is highly expressed in both UM and CM lines, suggesting that it may be a therapeutic target in a UM metastatic setting as it has been in CM models. Third, we found that RARg, PPARd, EAR2, RXRa, and TRa expressions could subdivide UM from CM. Previous studies of UM cancers identified key mutations in three genes: GNAQ, GNA11, and BRAF. We found unique NR expression profiles associated with each of these UM mutations. We then performed NR-to-NR and NR-to-genome expression correlation analyses to find potential NR-driven transcriptional programs activated in UM and CM. Specifically, RXRg controlled gene networks were identified that may drive melanoma-specific signaling and metabolism. ERRa was identified as a UM-defining NR and genes correlated with its expression confirm the role of ERRa in metabolic control. Given the plethora of available NR agonists, antagonists, and selective receptor modulators, pharmacologic manipulation of these NRs and their transcriptional outputs may lead to a more comprehensive understanding of key UM pathways and how we can leverage them for better therapeutic alternatives.

## Introduction

Uveal melanoma (UM) is the most common form of intraocular cancer in adults and comprises about 5% of all malignant melanoma diagnoses (1). UM tumors differ from cutaneous melanomas (CM) in that they arise from melanocytes of the choroid, ciliary body, and iris, defined as the uvea. Although there are effective therapeutic approaches for treating primary uveal tumors, more than 50% of patients exhibit hematogenous spread and metastatic disease, most often to the liver (~80–90% of cases) (2). Once UM disease has disseminated, therapeutic options are severely limited and average survival rates range from 2 to 8 months (3).

One of the major factors hampering development of therapeutic options for metastatic UM is the lack of discernible driver mutations. Unlike CMs, which frequently harbor BRAF^V600E^ or NRAS mutations, only ~5% of UMs (specifically, only UMs originating from the iris) exhibit BRAF^V600E^ mutations, and NRAS mutations are typically not observed in UM tumors (4). Recent mutational profiling studies of UM have identified mutually exclusive, activating mutations in two G protein coupled receptor alpha subunits, GNAQ and GNA11, in more than 80% of profiled UM tumors (5). These mutations appear to be relatively UM specific and are only found in about 5% of cases in other tumor types (6).

The nearly ubiquitous presence of the GNAQ and GNA11 mutations in UM suggests that they would make an effective therapeutic target, but functional studies of these mutations have noted them to be relatively weak oncoproteins that require other genetic alterations (including p53 and p16/CDK4/RB1 pathway inactivation) to transform immortalized melanocytes (7). Some success has been seen with targeting of downstream targets of GNAQ/GNA11 (specifically combined PKC and MEK inhibition), suggesting that indirect targeting of these mutations may be more effective (8). Recent advances have been made in understanding the underlying mechanism of the GPCR alpha subunit’s oncogenic activity, specifically the identification of the transcriptional coactivator yes-associated protein 1 (YAP 1) as a pro-proliferative oncogene and potential therapeutic target (9).

The nuclear receptor (NR) superfamily of transcription factors includes 48 members, most of which activate complex transcriptional programs via ligand binding (10). NRs regulate numerous physiological programs including developmental, homeostatic, proliferative, reproductive, and metabolic pathways (11). In a cancer context, NRs have been validated as pro-proliferative and oncogenic drivers in many tumor types including breast, ovarian, prostate, endometrial, and hematological malignancies (12, 13). In these diseases, NRs have proven to be effective therapeutic targets with numerous drugs targeting many NRs including estrogen receptor (ER) in breast, ovarian, and endometrial cancers, androgen receptor (AR) in prostate tumors, and glucocorticoid receptor (GR) in some hematological malignancies (14). In previously published work studying lung cancer, NR expression analysis has been successfully used to develop a prognostic signature for both survival and progression free survival and to identify potential therapeutic drug targets in pre-clinical models (15).

Nuclear receptors have also been noted as having tumor suppressive functions including VDR’s protective function in colon cancer (16); PPARg’s activation in reducing tumorigenicity in many cancer tissue types (17); TR4 and RARb as tumor suppressors in prostate (18, 19), and NUR77 and NOR1 as tumor suppressors in AML (20). Given the extensive roles that NRs play in the maintenance of normal development and physiology as well as the emerging understanding of NRs in oncogenic pathways, we set out to investigate how NR expression and activity may be leveraged to discover novel diagnostic, prognostic, and therapeutic alternatives in UM.

The expression and activity of NRs in UMs have been, to date, completely unstudied. To address this issue, we have used high-throughput qRT-PCR to profile the expression of the 48 members of the NR superfamily in various UM cell lines derived from both primary and metastatic lesions and in a normal melanocyte cell line. Based on these results, we report UM-specific NR expression patterns as well as pharmacologically targetable NR-regulated gene networks that could be driving proliferative or oncogenic signaling in UM.

## Materials and Methods

### Cell lines and RNA extractions

All UM cell lines were a kind gift from Dr. Jerry Niederkorn at UT Southwestern and were grown as originally described (21). CM cell lines were received from the NCI, NIH. Cell line identity was confirmed by fingerprinting and compared to standards when available. Primary adult human melanocytes were purchased from Cascade Biologics and were grown per the company’s instructions. Cell pellets were processed for RNA extraction using the RNeasy kit, according to the manufacturer’s protocol. Extracted RNA was quantified, aliquoted, and stored at −80°C and used to make the corresponding cDNA with Invitrogen’s (Carlsbad, CA, USA) First Strand kit.

### qRT-PCR and data analysis

Analysis of NR expression (mRNA) was performed in triplicate using the TaqMan-based efficiency-corrected cycle threshold method with 12.5 ng cDNA per reaction for 50 cycles in an ABI 7900HT sequence detection system (Applied Biosystems, Foster City, CA, USA) as previously described (22). NR mRNAs with cycle times >35 were determined to be below detection. Primer concentrations were 75 nM for 18S rRNA and 300 nM for NR primers; probes were added at 250 nM. The sequences of the validated primer/probe sets for the 48 human NRs are available at www.nursa.org under the rapid release tab. Universal cDNA standards generated from human adult RNA (BD Clontech, Palo Alto, CA, USA) were used for analysis of all receptors except CAR, FXRb, PXR, SHP, DAX-1, ERb, LRH-1, PNR, SF-1, and TLX, which were too limited in expression to use the universal RNA set. For these receptors, commercially available tissue-specific total RNA standards derived from cell lines or adult organ donors were used from liver, ovary, eye, adrenal, and brain, as appropriate. qRT-PCR data were analyzed using ABI instrument software SDS2.1. Baseline values of amplification plots were set automatically, and threshold values were kept constant to obtain normalized cycle times and linear regression data. Because PCR efficiencies for each receptor primer set vary, individual receptor PCR efficiencies were determined to permit receptor-to-receptor comparisons. PCR efficiencies were calculated from the slope of the resulting standard curves as reported previously (11). Normalized mRNA levels are expressed as arbitrary units and were obtained by dividing the averaged, efficiency-corrected values for NR mRNA expression by that for 18S RNA expression for each sample.

### Microarray data

All microarray data were obtained from published datasets available at the GEO Database (http://www.ncbi.nlm.nih.gov/gds/) (23–25). The data were generated using two different platforms, Affymetrix Human Genome U133 Plus 2.0 Array (63 UM tumors and nine NCI-60 melanoma cell lines) and Illumina HumanHT-12 V4.0 expression beadchip (three UM cell lines). Data were compiled and a consensus list of 17,700 unique genes was further analyzed as described in the manuscript text.

### Statistical methodologies

Dendrograms were generated using R Statistical Software. Analysis parameters included distance calculations using a Manhattan methodology and Ward’s method for cluster aggregation. Correlation coefficients for comparisons between data were calculated using Pearson Correlation. *p*-Values for comparisons between groups of measurements were performed using Student’s *t*-tests. Bonferroni Corrections were used to account for multiple-hypothesis testing as appropriate.

### Gene ontology analysis

Correlation coefficients (Pearson) were calculated for each gene and the NR in question (either ERRa or RXRg). Lists were then culled to retain the most significantly positively correlated genes (cutoff of *r* > 0.6 was used). Culled lists were input into gene ontology (GO) analysis tool GOrilla and GO analyses were performed as described (26).

## Results

### NR expression in melanomas

**Figure 1:**
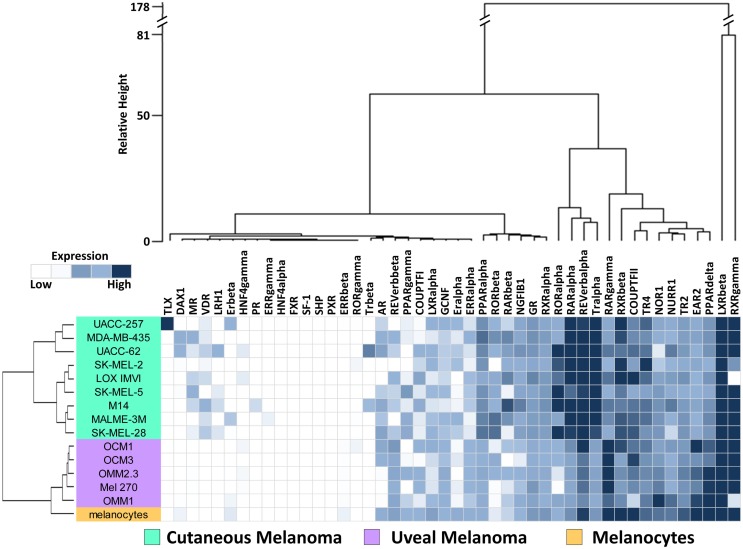
**Nuclear receptor expression in melanomas**. Clustered heat map representation of mRNA expression levels of the 48 human NRs in a panel of human uveal melanoma (*n* = 5) and cutaneous melanoma (*n* = 9) cell lines as measured by qRT-PCR. Additionally, a melanocyte was included as a normal comparator. Unsupervised hierarchical clustering (Distance calculations: Manhattan, Aggregation method: Ward’s) was performed on the NRs (columns) and cell line samples (rows, colored according to key). qRT-PCR data were quantified using standard curves and normalized to 18S as described in Section “Materials and Methods.” Data are color coded such that relatively highly expressed NRs are dark blue while lowly or unexpressed receptors (cycle time >35) are white.

To investigate the expression levels of the human NR superfamily (*n* = 48) in UM, we performed high-throughput qRT-PCR expression analysis across a cell line panel consisting of five UM cell lines, nine CM cell lines from the NCI-60 (27), and one primary melanocyte control (Figure 1). A heat map was generated to display the results and it was seen that several receptors including SF-1, SHP, TLX, PR, and HNF4a are either expressed at very low levels or completely unexpressed across the panel, suggesting that they do not play a large role in either CM or UM. Other receptors, such as COUPT-FII, LXRb, and RXRg, were found to be strongly expressed across all the samples analyzed. GR, NOR1, NURR1, PPARa, TR2, and TR4 were also expressed in all samples, but at more moderate levels.

Data quality was assessed by comparison to 63 previously published microarray profiles generated from patient UM samples (Figure S1 in Supplementary Material) (24). In this comparison, it was seen that NRs unexpressed in the qRT-PCR dataset reported here were also unexpressed in the patient dataset. Likewise, NRs found highly expressed in the qRT-PCR dataset were generally well expressed in the UM patient samples. Examples of these NRs are shown on Figure S1 in Supplementary Material. Overall, Spearman rank correlation of the expression levels of the 48 NRs between the two datasets was found to be 0.619 (*p* < 0.0001), suggesting that the data presented here for UM lines are representative of findings in clinical UM samples.

### Hierarchical clustering of cell lines by NR expression

To visualize relationships within the dataset, unsupervised hierarchical clustering analyses were performed on the qRT-PCR dataset for both the 13 cell lines and the 48 NRs (Figure 1). For these analyses, distance matrices were calculated using the Manhattan distance methodology and clusters were aggregated based on Ward’s method. First, we observed that the expression of the 48 NRs can properly segregate the UM lines from the CM lines. The known mutational spectrum of CM and UM would suggest that these two types of melanoma differ from each other, and the unique NR expression signatures seen here, support this idea. As would be expected, NR expression does subdivide the normal melanocyte from the tumor cell lines, but interestingly places the melanocyte in closer proximity to the uveal cluster.

Analysis of the clusters generated by aggregation of the NR probes found several levels of distinctions. Primary subdivisions include a large group of receptors that are either lowly expressed or fairly uniformly expressed, and a second group of receptors that are differentially expressed between CM and UM samples. Interestingly, two NRs (LXRb and RXRg) were particularly distinct from the other NRs in the dataset due to their exceptionally high expression. Previously published work from our group noted that RXRg expression is very high in CM cell lines while essentially unexpressed in every other tissue type in the NCI-60 cancer cell line panel (27). High levels of RXRg expression are similarly seen in UM. When the UM samples were clustered together with the previously published NCI-60 NR expression data, it was found that RXRg expression defined a “melanoma cluster,” which contained both UM and CM samples (Figure S2 in Supplementary Material).

### Differential expression of NRs in UM compared to CM

**Figure 2:**
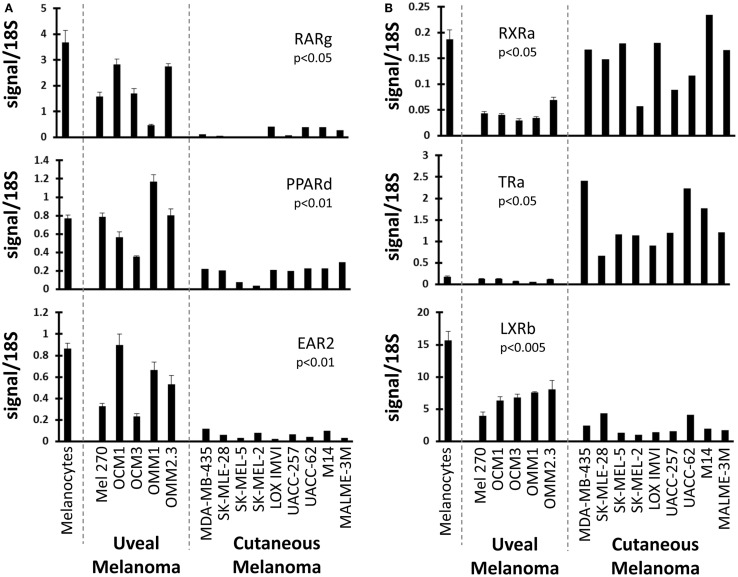
**Uveal versus cutaneous melanoma NR expression patterns**. Patterns of mRNA expression across the cell line panel for select receptors. *p*-Values are shown for the comparison of the expression level of the indicated nuclear receptor between uveal and cutaneous melanoma cell lines. Calculations were performed using a Student’s *t*-test and were Bonferroni-corrected for to account for multiple-hypothesis testing. **(A)** Three receptors (RARg, PPARd, and EAR2) are lost in cutaneous melanoma but retained in the uveal melanoma samples (versus melanocyte expression). **(B)** (Top) RXRa is lost in uveal melanoma samples. (Middle) TRa is overexpressed by cutaneous melanomas. (Bottom) LXRb expression is higher in uveal melanoma samples.

We next examined NRs differentially expressed between UM and CM (Figure 2). First, three NRs (RARg, PPARd, and EAR2) were found to have significantly lower expression levels in CM than in UM cell lines. Comparison to the melanocyte control suggested that the UM samples had retained “normal” expression of these receptors while expression had been lost in the CM samples. Conversely, it was found that RXRa expression was lower in UM than in CM or in the melanocyte control, suggesting UM had specifically lost RXRa expression. Analysis of TRa levels found that UM lines retained expression of this NR similar to that of the melanocyte, but that expression of TRa was significantly higher in CM. By contrast, REVerb an expression appears to be lost specifically in UM. As was previously mentioned, LXRb is highly expressed across all melanoma samples, but is expressed significantly higher in UM samples. This finding is particularly notable since LXRb agonists have been shown to reduce proliferative and metastatic potential in CM pre-clinical mouse models (28).

### Clusters of co-expressed NRs

**Figure 3:**
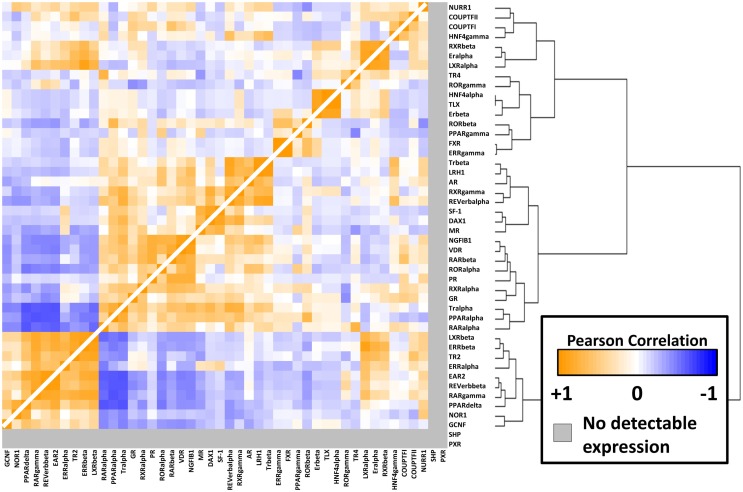
**NR-to-NR pairwise correlation comparisons**. Pairwise Pearson correlation coefficient (PCC) analysis of the nuclear receptors. Hierarchical clustering analysis was performed as in Figure 1. Positive correlations are depicted in orange with the strongest intensities corresponding to higher correlations (0.95 was the highest pairwise correlation). Similarly, negative correlations are depicted in blue tending toward white as they become less intense. The range of positive correlations (0–0.95) was greater than the range of negative correlations (0 to −0.64).

To better understand the relationships between the NRs themselves within the melanoma panel, we calculated correlation coefficients (performed as before) for all possible pairwise combinations of the 48 NRs. The results of this unsupervised clustering analysis are shown as a heat map in Figure 3. Several clusters of strongly positive correlations could be seen, including a cluster containing receptors identified as differentially regulated between CM and UM. The CM-specific cluster included EAR2, REV-ERb, RARg, NOR1, and GCNF while the UM-specific cluster contained LXRb, ERRb, TR2, and ERRa. These pockets of strong correlation suggest transcriptional and/or functional interconnections within these receptor subgroups and within specific melanoma subtypes.

### Differential expression of NRs across UM

**Figure 4:**
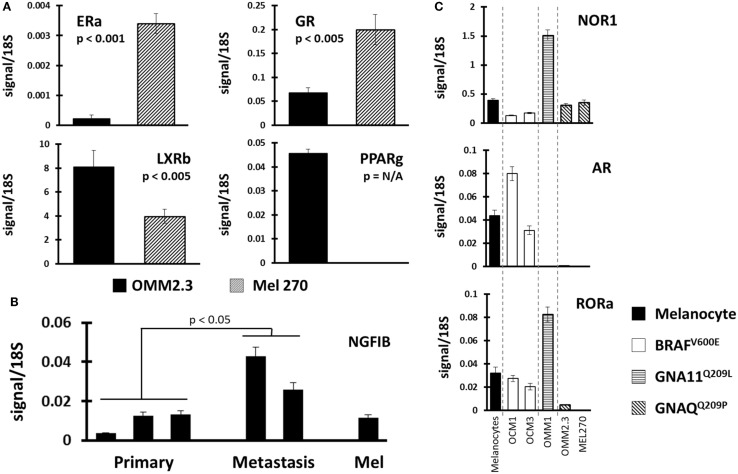
**Identification of NR expression patterns within uveal melanoma subtypes**. **(A)** Comparisons were made between replicate data generated from UM cell line pair OMM2.3 (metastasis-derived) and MEL270 (primary-derived) with *p*-values calculated through a Bonferroni-corrected Student’s *t*-test. ERa and GR expressions were lost in the metastatic line (top) while LXRb and PPARg expressions were higher in the metastasis (bottom). **(B)** NGFIB expression was found higher in the metastasis-derived UM cell lines versus the primary-derived UM lines (*p*-value from corrected *t*-test). **(C)** Receptor expression was compared between the different mutational states of UM. (Top) NOR1 was found overexpressed in the GNA11 mutant, (middle) AR expression was retained in the BRAF mutants, and (bottom) RORa expression was lost in the GNAQ mutant cell lines.

Finally, we compared NR profiles across the different UM cell lines (Figure 4). Because UM metastatic disease is essentially fatal, we were particularly interested in identifying metastasis-specific NR expression patterns. Initially, we chose to compare NR expression in the cell line pair MEL270 (primary) and OMM2.3 (metastasis), both of which were derived from the same patient. Several genes were found differentially expressed between the pair with the most significant, liganded NRs highlighted in Figure 4A. ERa and GR expressions were found to be lost in the metastasis-derived OMM2.3 versus the primary MEL270. Conversely, LXRb and PPARg expressions were either significantly lower (LXRb) or completely undetected (PPARg) in the primary while robust expression was seen in the metastatic line, suggesting that these genes were up-regulated during the metastatic process and may be essential for retention of proliferative capacity or for survival at distant anatomical sites.

Within the UM panel, three cell lines were derived from primary tumors (OCM3, MEL270, and OCM1) and two cell lines were derived from metastatic lesions (OMM1 and OMM2.3). Comparison between these two groups found that NGFIB was up-regulated in the metastatic lines versus the primary tumor lines and the melanocyte control line (Figure 4B). There were several other NRs that were up-regulated in the metastatic group, but the small sample size and heterogeneity among primary tumor lines precluded statistical significance.

Examples of all three of the key UM mutations are represented in the cell line panel (Table 1). Because GNAQ, GNA11, and BRAF mutations are mutually exclusive in clinical samples (5), we identified potentially mutation-specific NR expression patterns (Figure 4C). First, we found that the GNA11 mutant cell line OMM1 showed significant overexpression of NOR1 versus the rest of the panel. Comparisons between the BRAF mutant UM lines OCM1 and OCM3 and the rest of the panel found that the BRAF mutants retained expression levels of AR comparable to the melanocyte while the other cell lines had lost AR expression. Finally, it was found that both OMM2.3 and MEL270 (GNAQ mutants) had almost completely lost RORa expression while the other cell lines maintained RORa expression at levels comparable to the primary melanocyte line.

**Table 1:** **Mutation status of cell lines studied**.

Cell line	Gq mutant	G11 mutant	BRAF mutant
Melanocytes	WT	WT	WT
OCM1	WT	WT	V600E
OCM3	WT	WT	V600E
OMM1	WT	Q209L	WT
OMM2.3*	Q209P	WT	WT
Mel270*	Q209P	WT	WT
LOXIMVI	WT	WT	V600E
M14	WT	WT	V600E
MALME-3M	WT	WT	V600E
MDA-MB-435	WT	WT	V600E
SK-MEL-2	WT	WT	V600E
SK-MEL-28	WT	WT	V600E
SK-MEL-5	WT	WT	V600E
ACC-257	WT	WT	V600E
UACC-62	WT	WT	V600E

*Indicates cell lines from same patient.

*The cell lines used in this study are listed, along with their mutation status for GNAQ^Q209P^ (Gq), GNA11^Q209L^ (G11), and BRAF^V600E^ (BRAF). WT, wild type*.

### NR-driven gene networks in UM

Finally, because NR activity has been largely unstudied in UM, we examined publically available microarray profiles generated from both UM and CM cell lines (23, 25) to identify gene networks that might be NR-regulated in the melanoma context. For these analyses, we selected two liganded NRs (ERRa and RXRg) that are expressed across the melanoma panel and that correlated strongly with our qRT-PCR data for these same cell lines (*r* = 0.82 for ERRa and 0.7 for RXRg). Lists of microarray probes positively correlated with either ERRa or RXRg expression patterns were generated, culled to the top 400 genes correlating with each receptor (Table S1 in Supplementary Material), and subjected to gene ontology (GO) analysis using publically available GO analysis tool Gorilla (26). Top GO terms associated with ERRa and RXRg are shown in Tables 2 and 3, respectively. For RXRg (the main differentiator between melanomas and other cancers), top GO terms included numerous lipid and basal metabolism associated functions as expected (10), suggesting RXRg may be regulating these functions within a melanoma context. Furthermore, four GO terms associated with RXRg pertained to peroxisome activity [RXRs and PPARs have a well-established relationship (10)], suggesting RXRg could acquire activities within a melanoma setting. One of the genes defining the UM cluster was ERRa, a known regulator of metabolic pathways (29). Predictably, 11 of the top 12 GO terms involved metabolic regulation, indicating that ERRa may contribute to regulation of metabolism in UM, a role that may be pharmacologically targetable via inverse agonists of ERRa.

**Table 2:** **ERR alpha associated gene ontology (GO) terms**.

GO term	Description	*p*-Value	FDR *q*-value
GO:0044238	Primary metabolic process	2.35E-05	3.07E-01
GO:0071704	Organic substance metabolic process	5.93E-05	3.88E-01
GO:0044260	Cellular macromolecule metabolic process	8.73E-05	3.81E-01
GO:0044237	Cellular metabolic process	1.03E-04	3.37E-01
GO:0043170	Macromolecule metabolic process	1.93E-04	5.05E-01
GO:0090304	Nucleic acid metabolic process	2.28E-04	4.98E-01
GO:0006139	Nucleobase-containing compound metabolic process	2.59E-04	4.84E-01
GO:0016070	RNA metabolic process	4.45E-04	7.28E-01
GO:0046483	Heterocycle metabolic process	4.85E-04	7.05E-01
GO:0006725	Cellular aromatic compound metabolic process	5.08E-04	6.65E-01
GO:0006366	Transcription from RNA polymerase II promoter	6.71E-04	7.98E-01
GO:0009058	Biosynthetic process	9.59E-04	1.00E + 00

*Gene ontology descriptions of genes positively correlated to ERR alpha expression levels in UM are shown, along with the corresponding *p*-value and false discovery rate *q*-values*.

**Table 3:** **RXR gamma associated gene ontology (GO) terms**.

GO term	Description	*p*-Value	FDR *q*-value
GO:0044255	Cellular lipid metabolic process	4.39E-06	5.75E-02
GO:0006629	Lipid metabolic process	1.36E-05	8.89E-02
GO:0008610	Lipid biosynthetic process	3.67E-05	1.60E-01
GO:0006631	Fatty acid metabolic process	3.87E-05	1.27E-01
GO:0032787	Monocarboxylic acid metabolic process	1.20E-04	3.15E-01
GO:0071616	Acyl-coA biosynthetic process	1.26E-04	2.75E-01
GO:0035384	Thioester biosynthetic process	1.26E-04	2.36E-01
GO:0019752	Carboxylic acid metabolic process	2.26E-04	3.70E-01
GO:0006082	Organic acid metabolic process	2.81E-04	4.09E-01
GO:0032868	Response to insulin	3.98E-04	5.21E-01
GO:0006625	Protein targeting to peroxisome	5.38E-04	6.40E-01
GO:0072663	Establishment of protein localization to peroxisome	5.38E-04	5.87E-01
GO:0072662	Protein localization to peroxisome	5.38E-04	5.42E-01
GO:0043436	Oxoacid metabolic process	5.56E-04	5.20E-01
GO:0006790	Sulfur compound metabolic process	6.29E-04	5.48E-01
GO:0043574	Peroxisomal transport	6.57E-04	5.38E-01
GO:0006633	Fatty acid biosynthetic process	8.21E-04	6.32E-01

*Gene ontology descriptions of genes positively correlated to RXR gamma expression levels in UM are shown, along with the corresponding *p*-value and false discovery rate *q*-values*.

## Discussion

In this study, we measured expression levels of the 48 human NRs by qRT-PCR across a panel of UM cell lines and a normal melanocyte control to expand on our original work across the NCI-60 panel. We demonstrated that UM (like CM) is distinguished from other cancer cell lines by high expression of RXRg, an NR that we previously reported separates melanomas from other cancers. Furthermore, UM and CM can be differentiated based solely on their NR expression profiles with several NRs differentially expressed between the two (including RARg, PPARd, EAR2, RXRa, and TRa). Our results confirm the distinction of UM and CM as separate diseases in line with their differing mutational profiles. We also examined whether there are NRs preferentially expressed in the different mutational subtypes of UM (GNA11^Q209L^, BRAF^V600E^, and GNAQ^Q209P^) and identified receptors (NOR1, AR, and RORa, respectively) exhibiting mutation-specific expression patterns.

Because of the particular importance of metastases within UM, we compared primary-derived and metastasis-derived UM cell lines to identify NRs that might be playing specific roles within the metastatic context. We discovered that NGFIB was up-regulated in UM metastatic cell lines versus primary-derived UM cell lines. Although this was the only NR that met our statistical threshold, it is notable that two other members of the NR4 family (NOR1 and NURR1) also showed generally higher expression in the metastasis-derived UM cell lines. The role of NR4 family members in cancer is controversial as it has been noted to be pro-proliferative in some contexts and tumor suppressive in others (20). One other notable NR that trended toward higher expression in the metastasis-derived uveal lines was PPARg, which has been noted in many cancer models to be anti-proliferative upon ligand activation (17).

Finally, we examined NR-to-NR correlation patterns as well as NR-to-genome correlation patterns to identify receptors that might be interacting with each other and to identify networks of genes that certain, key NRs might be controlling within the UM context. ERRa was one of the NRs that differentiated the UM cluster from the CM cluster, and is particularly important given its role as a therapeutic target in other cancers (particularly breast cancer) and the growing availability of ERRa-targeted therapeutics (30) including inverse agonists, which lower the receptor’s constitutive activity.

Analogous studies conducted previously by our group and others have successfully utilized NR expression profiles to subdivide between different cancer types. It has been demonstrated that expression profiles of the 48 NRs alone can properly distinguish between cancers of vastly different origins (within the NCI-60 panel) (27), between small-cell and non-small-cell lung cancers (15), and between different types of thyroid cancers (31). Here, we further add the differentiation by NRs between UM and CMs, suggesting that the use of NR expression patterns may be broadly applicable as a tool to categorize different cancers and histological groupings. Given their key roles in regulation of global cellular signaling processes and in cellular development pathways, it is not surprising that NRs play such a central role to the identities of these cell types, even within a dedifferentiated cancer state.

It is of significant interest that several NRs are differentially expressed between UM and CM tumors. Particularly, the findings RARg, PPARd, EAR2, TRa, and LXRb that are retained in UM at levels comparable to the melanocyte while being reduced or lost in CM suggests that there may be opportunities for NR-directed therapeutic interventions in UM that are not available in CM due to CM-specific loss of these receptors. It is also worth noting that the UM-specific loss of RXRa expression. Because RXRa is a heterodimeric binding partner for many NRs, lower RXRa expression might suggest indirect downregulation of NR signaling in UM tumors.

It has recently been reported that metastatic CM can be inhibited by administration of LXRb agonists in pre-clinical models of CM (28). Here, we report that LXRb receptor levels are even higher in UM samples than the levels observed in CM samples. Given the known differences between UM and CM tumors, it will be important for future studies to examine whether or not LXRb-directed therapies or other NR ligand strategies will be effective in controlling UM metastatic disease *in vivo*.

Melanoma is an aggressive, highly metastatic disease that is notoriously difficult to treat using standard cytotoxic agents (32). Mechanistic studies have found that CM cells achieve their hallmark chemoresistance through genome-scale reprogramming of proliferation and survival pathways during disease progression. Given these findings, it is not surprising that many modern therapeutic strategies involve induction of wholesale changes in the transcriptome of CM cells through epigenetic modulation to overcome these anti-apoptotic and pro-proliferative pathways (33, 34). Although much work has been done in CM, far fewer studies have investigated ocular-derived UMs where metastatic disease is equally as fatal (3).

Mutational profiling of UM has identified mutually exclusive, UM specific, activating mutations in two paralogs (GNAQ and GNA11) in more than 80% of UM cases. Although these mutations would seem obvious targets for therapeutic intervention, GNAQ/11 mutations have not been amenable to therapeutic development in UM and recent work has instead focused on inhibiting downstream events and gene networks driven by these mutations (9, 35). As an example, combination therapy with inhibitors of GNAQ/11 downstream target protein kinase C (PKC) and MEK has been shown to inhibit the *in vitro* growth of GNAQ/11 UM mutant cell lines (8). Another recently identified downstream target of GNAQ/11 mutants is YAP1 and a YAP1 inhibitor, verteporfin, has also been shown to be effective inhibiting UM growth in xenograft models (35). However, as was pointed out in a recent preview opinion from Field and Barbour (36), it is important to note that these inhibitors alone will likely be insufficient for treating UM metastases as GNAQ/11 mutations are only weakly oncogenic being unable to transform immortalized melanocytes without additional, cooperating mutations (7). Recent clinical trial results using the MEK inhibitor selumetinib in metastatic UM patients underscore their opinion as there was no overall survival benefit (37).

As transcription factors, activated NRs are extremely effective in eliciting widespread physiologic changes in cells through alteration of the transcriptional output and architecture of the genome (38). One of the most striking examples of ligand-mediated NR activity comes from studies of estradiol’s effects on the transcriptome of an ER-positive breast cancer cell line. They report nearly 23,000 transcripts (equivalent to more than 25% of total cellular transcriptomic output) that are altered during ER activation (39). Other ligand/receptor combinations known to elicit broad-scale expression changes include mifepristone/progesterone receptor in endometrial tissue (40) and T0901317/liver X receptors in the human monocytic cell line THP-1 (41). Defining which NRs might be playing a role in transcriptional reprogramming during UM onset and progression, as we have begun to do here, should catalyze a better understanding and targeting of this disease. Overall, it will be interesting to see how NR expression patterns correlate with clinical disease progression in the future release of the UM TCGA dataset, to then design NR-driven therapeutic strategies.

## Conflict of Interest Statement

The authors declare that the research was conducted in the absence of any commercial or financial relationships that could be construed as a potential conflict of interest.

## Supplementary Material

The Supplementary Material for this article can be found online at http://journal.frontiersin.org/article/10.3389/fendo.2015.00093
