# Supplementary material for: A Subset of Nuclear Receptors are Uniquely Expressed in Uveal Melanoma Cells
Source: Front Endocrinol (Lausanne). 2015 Jul 7;6:93. doi: 10.3389/fendo.2015.00093 (PMC4493406; doi:10.3389/fendo.2015.00093)
Supplement: Supplementary file 1 [file Data_Sheet_1.PDF]

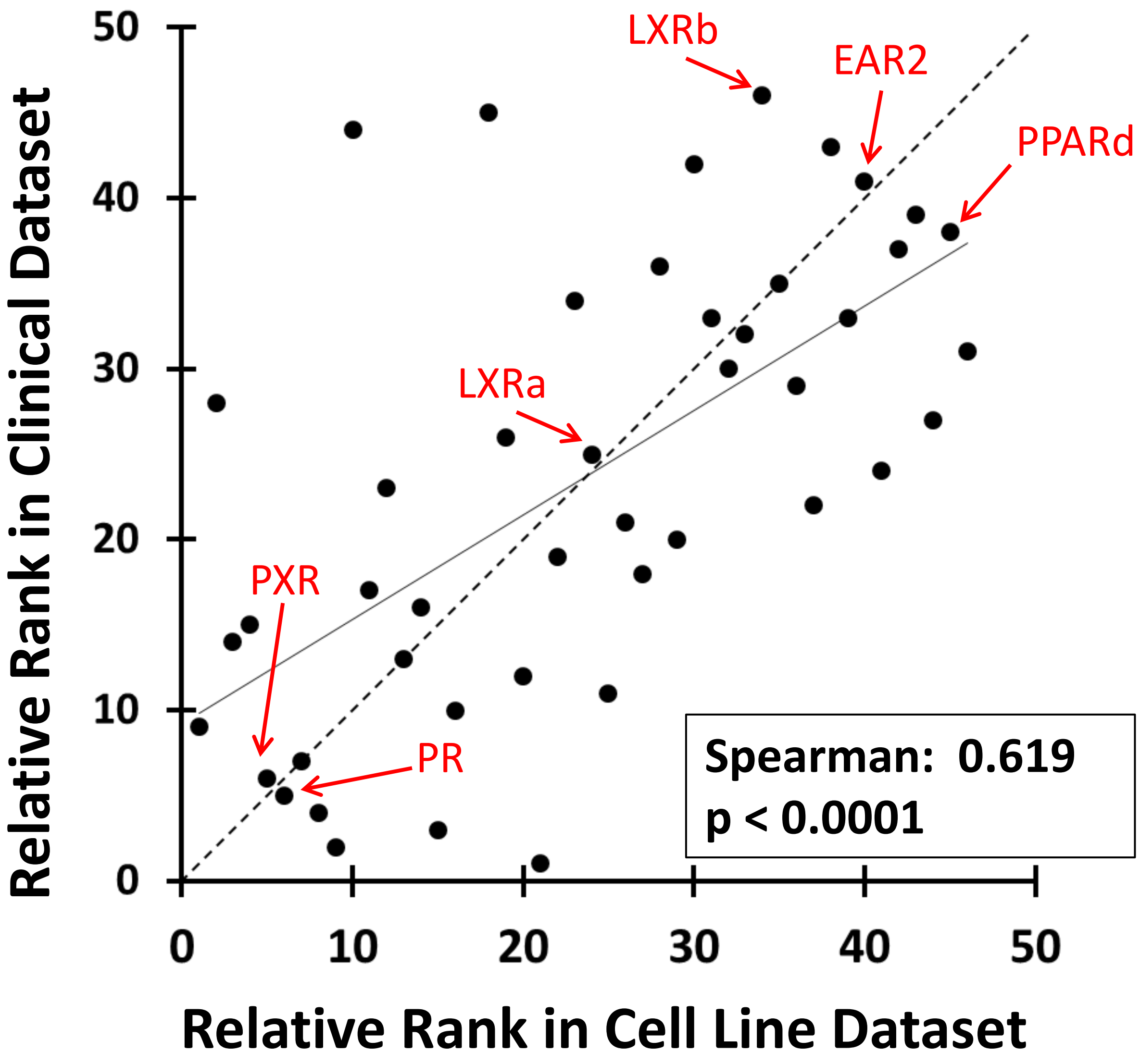

Perfect Concordance – solid line (———)

Experimental Result – dashed line (··········)

Fig. S1. Comparison of uveal melanoma qRT-PCR results with microarrays performed on uveal melanoma patient samples (n = 63). The relative rank of each NR in each dataset was compared by spearman correlation (r = 0.619). It was found that receptors lost in one dataset were typically lost in the other dataset while those retained or overexpressed in one dataset were typically also retained or overexpressed in the second dataset.

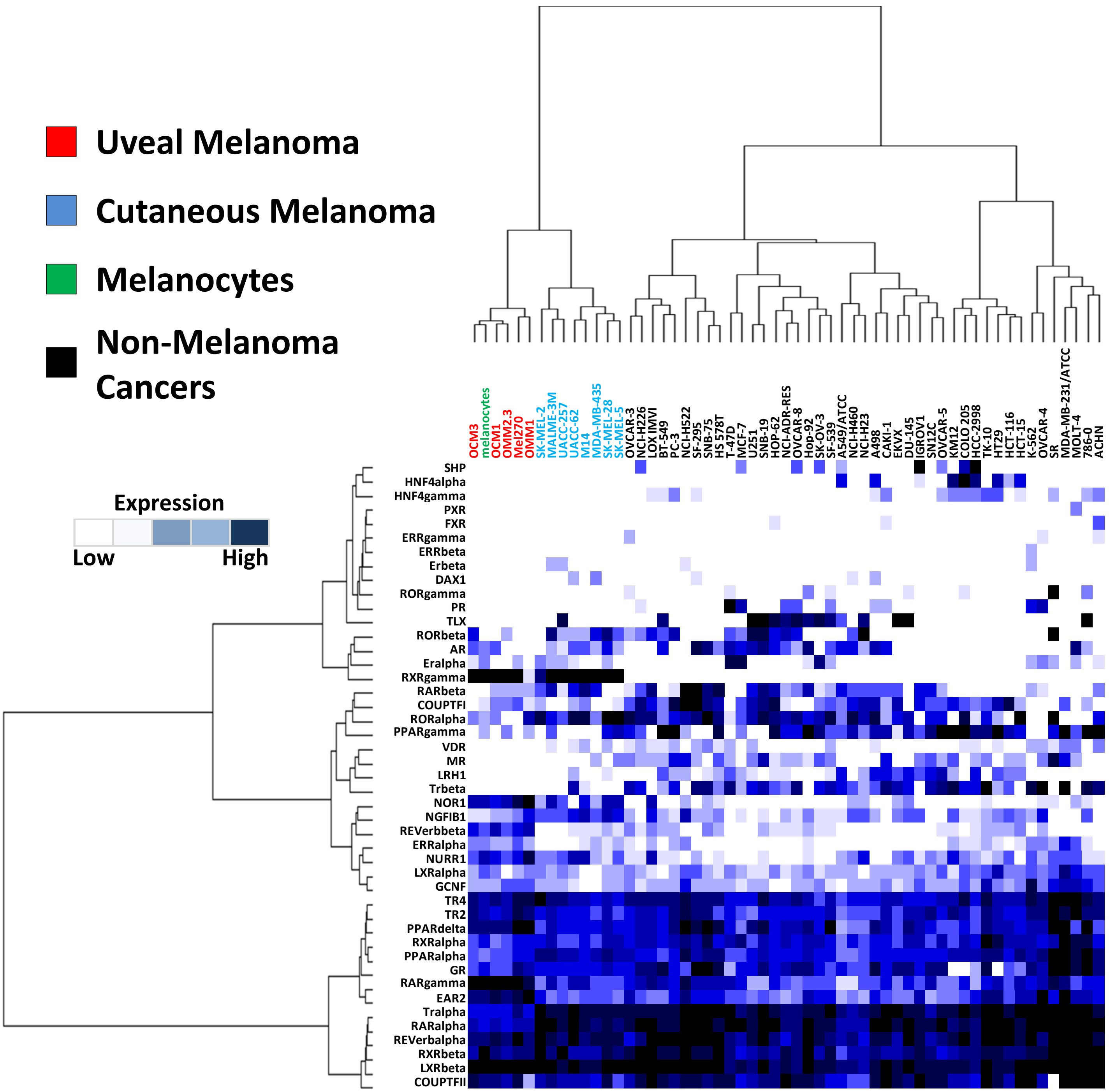

Fig. S2. Clustering by NR expression of uveal melanoma cell lines with the NCI-60 cancer panel. Previously published NR expression data qRT-PCR on the NCI-60 cancer cell line panel was combined with data presented here. Clustering revealed first that both types of melanoma were distinct from the rest of the non-melanoma cancers (largely due to RXRg expression). Within the melanoma cluster, clear distinction could be seen between uveal and cutaneous melanoma samples.
